# Prolactin-Releasing Peptide Contributes to Stress-Related Mood Disorders and Inhibits Sleep/Mood Regulatory Melanin-Concentrating Hormone Neurons in Rats

**DOI:** 10.1523/JNEUROSCI.2139-21.2022

**Published:** 2023-02-01

**Authors:** Szilvia Vas, Rege S. Papp, Katalin Könczöl, Emese Bogáthy, Noémi Papp, Csaba Ádori, Máté Durst, Klaudia Sípos, Klementina Ocskay, Imre Farkas, Flóra Bálint, Szilamér Ferenci, Bibiána Török, Anita Kovács, Evelin Szabó, Dóra Zelena, Krisztina J. Kovács, Anna Földes, Erzsébet Kató, László Köles, György Bagdy, Miklós Palkovits, Zsuzsanna E. Tóth

**Affiliations:** ^1^Department of Pharmacodynamics, Semmelweis University, Budapest, 1089, Hungary; ^2^MTA-SE Neuropsychopharmacology and Neurochemistry Research Group, Semmelweis University, Budapest, 1089, Hungary; ^3^Human Brain Tissue Bank and Laboratory, Semmelweis University, Budapest, 1094, Hungary; ^4^Laboratory of Neuroendocrinology and In Situ Hybridization, Department of Anatomy, Histology and Embryology, Semmelweis University, Budapest, 1094, Hungary; ^5^Department of Neuroscience, Karolinska Institutet, Stockholm, 17177, Sweden; ^6^Laboratory of Reproductive Neurobiology, Institute of Experimental Medicine, Eötvös Loránd Research Network, Budapest, 1083, Hungary; ^7^Laboratory of Endocrine Neurobiology, Institute of Experimental Medicine, Eötvös Loránd Research Network, Budapest, 1083, Hungary; ^8^Laboratory of Molecular Neuroendocrinology, Institute of Experimental Medicine, Eötvös Loránd Research Network, Budapest, 1083, Hungary; ^9^Laboratory of Behavioral and Stress Studies, Institute of Experimental Medicine, Eötvös Loránd Research Network, Budapest, 1083, Hungary; ^10^Institute of Physiology, Medical School, University of Pécs, Centre for Neuroscience, Szentágothai Research Center, Pécs, 7624, Hungary; ^11^Department of Oral Biology, Semmelweis University, Budapest, 1089, Hungary; ^12^Department of Pharmacology and Pharmacotherapy, Semmelweis University, Budapest, 1089, Hungary; ^13^NAP2-SE New Antidepressant Target Research Group, Budapest, 1085, Hungary

**Keywords:** GABA(A) receptor, MCH, mental disorders, sleep, stress, suicide

## Abstract

Stress disorders impair sleep and quality of life; however, their pathomechanisms are unknown. Prolactin-releasing peptide (PrRP) is a stress mediator; we therefore hypothesized that PrRP may be involved in the development of stress disorders. PrRP is produced by the medullary A1/A2 noradrenaline (NA) cells, which transmit stress signals to forebrain centers, and by non-NA cells in the hypothalamic dorsomedial nucleus. We found in male rats that both PrRP and PrRP-NA cells innervate melanin-concentrating hormone (MCH) producing neurons in the dorsolateral hypothalamus (DLH). These cells serve as a key hub for regulating sleep and affective states. *Ex vivo*, PrRP hyperpolarized MCH neurons and further increased the hyperpolarization caused by NA. Following sleep deprivation, intracerebroventricular PrRP injection reduced the number of REM sleep-active MCH cells. PrRP expression in the dorsomedial nucleus was upregulated by sleep deprivation, while downregulated by REM sleep rebound. Both in learned helplessness paradigm and after peripheral inflammation, impaired coping with sustained stress was associated with (1) overactivation of PrRP cells, (2) PrRP protein and receptor depletion in the DLH, and (3) dysregulation of MCH expression. Exposure to stress in the PrRP-insensitive period led to increased passive coping with stress. Normal PrRP signaling, therefore, seems to protect animals against stress-related disorders. PrRP signaling in the DLH is an important component of the PrRP's action, which may be mediated by MCH neurons. Moreover, PrRP receptors were downregulated in the DLH of human suicidal victims. As stress-related mental disorders are the leading cause of suicide, our findings may have particular translational relevance.

**SIGNIFICANCE STATEMENT** Treatment resistance to monoaminergic antidepressants is a major problem. Neuropeptides that modulate the central monoaminergic signaling are promising targets for developing alternative therapeutic strategies. We found that stress-responsive prolactin-releasing peptide (PrRP) cells innervated melanin-concentrating hormone (MCH) neurons that are crucial in the regulation of sleep and mood. PrRP inhibited MCH cell activity and enhanced the inhibitory effect evoked by noradrenaline, a classic monoamine, on MCH neurons. We observed that impaired PrRP signaling led to failure in coping with chronic/repeated stress and was associated with altered MCH expression. We found alterations of the PrRP system also in suicidal human subjects. PrRP dysfunction may underlie stress disorders, and fine-tuning MCH activity by PrRP may be an important part of the mechanism.

## Introduction

Chronic or repeated exposure to stress may cause serious mental health conditions, such as anxiety, post-traumatic stress disorder (PTSD), and major depressive disorder (Smoller, 2016). Dysregulation of the hypothalamus-pituitary-adrenal (HPA) axis, the stress axis, as well as mood and sleep disturbances are typical symptoms in patients, which develop by unknown mechanisms (Goldstein and Walker, 2014; Bao and Swaab, 2019).

Serotonin and noradrenaline (NA) reuptake inhibitor (SNRI) antidepressants are commonly used to treat major depressive disorder, anxiety, and PTSD, although treatment resistance to conventional SNRIs occurs frequently (Voineskos et al., 2020). Neuropeptides, which can modulate neurotransmitter levels in the brain (neuromodulators), may offer alternative antidepressant targets (Mantas et al., 2022). Here, we designate prolactin-releasing peptide (PrRP)/prolactin-releasing hormone (PRLH) as a novel candidate known to be involved in the regulation of stress, sleep, and mood (Dodd and Luckman, 2013). PrRP may act in part by affecting melanin-concentrating hormone (MCH) producing cells in the dorsolateral hypothalamus (DLH). Our hypothesis is based on the following.

PrRP neurons are in a decisive position for transmitting stress-related signals toward higher brain centers and influence the efficacy of noradrenergic transmission. The adaptive response of the HPA axis to homeostatic stressors and peripheral inflammation depends on the integrity of the ascending pathway from the medullary A1/A2 noradrenergic cell groups (Pacak et al., 1995). PrRP is expressed exclusively in the A1/A2 cell groups (Chen et al., 1999) and the hypothalamic dorsomedial nucleus (DM) (Dodd and Luckman, 2013). PrRP cells respond to various psychogenic stressors, such as conditioned-fear stimuli, foot shock, restraint, inflammatory stress, etc. (Ohiwa et al., 2007; Dodd and Luckman, 2013). PrRP-deficient mice fail to increase plasma ACTH following reexposure to a conditioned fear stimulus (Yoshida et al., 2014). Serving key role in stress, PrRP stimulates the release of ACTH (Maruyama et al., 2001) and corticosterone (Mera et al., 2006) *in vivo*, and substantially potentiates the ACTH-stimulatory effect of NA (Maruyama et al., 2001).

PrRP also affects the sleep–wake cycle, although the available literature is contradictory (Zhang et al., 2001; Lin et al., 2002). *Ex vivo* studies suggest that the reticular nucleus of the thalamus may mediate some effects of PrRP on the sleep–wake cycle (Lin et al., 2002), where PrRP alias PRLH receptor (PRLHR) expression is high, but PrRP axons are absent (Dodd and Luckman, 2013). In contrast, PrRP fibers innervate the DLH (Dodd and Luckman, 2013). The DLH is populated by MCH cells (Verret et al., 2003), which cells are considered a key regulatory hub of sleep and mood, moreover, participate in the control of stress-response (Smith et al., 2006; Diniz and Bittencourt, 2017; Bandaru et al., 2020). A series of data highlights MCH dysfunction underpinning the pathophysiology of anxiety, depression, and PTSD (Chaki et al., 2005; Chung et al., 2011; Torterolo et al., 2015; Concetti et al., 2020). Although antagonists of MCH1 receptor (MCH1R) have shown promise as antidepressant drugs (Chung et al., 2011; Johansson and Löfberg, 2015), the exact mechanism of how MCH contributes to stress-related mental disorders remains to be elucidated. MCH neuronal activity during acute stress (foot shock) was necessary for subsequent coping with contextual fear (Izawa et al., 2019) and for cued fear extinction (Concetti et al., 2020). Controversially, the administration of MCH in a repeated stress paradigm (learned helplessness [LHe]) impaired the animals' ability to cope adequately with a second foot shock, resulting in helpless behavior (Urbanavicius et al., 2019). Thus, it appears that proper control of MCH signaling is critical in preventing the adverse effects of chronic/repeated stress.

We applied an array of histologic and electrophysiological methods, as well as depression models, and analyzed human PrRP receptor expression in the DLH of suicidal subjects. Our data reveal that the PrRP system may contribute to the development of stress-related mental disorders because of defects in PrRP signaling caused by chronic/repeated stress. We propose that PrRP regulates the function of MCH neurons; furthermore, PrRP signaling in the DLH is impaired in animal models of depression and in suicidal humans.

## Materials and Methods

### Animals

The experimental animals were male Wistar rats (Toxi-Coop), housed and kept under standard laboratory conditions with 12/12 h light/dark cycle (lights on at 6:00 A.M., unless otherwise stated). We used males to exclude sex effects on the data, as PrRP expression varies depending on female gonadal hormone levels (Tóth et al., 2008). Rats were 10-12 weeks old, unless otherwise indicated. All experiments were performed in accordance with the National Institutes of Health's “Principles of Laboratory Animal Care” (NIH Publications No. 85-23, revised 1985), international standards (European Community Council Directive, 86/609/EEC, 1986, 2010), and specific national laws (the Hungarian Governmental Regulations on animal studies 40/2013). The experiments were approved by the National Scientific Ethical Committee on Animal Experimentation, the Ethical Review Board of the Semmelweis University (PE/EA/1563-7/2017, PE/EA/850-2/2016, PTE/84 033-6/2020), and the Animal Welfare Committee of the Institute of Experimental Medicine (PE/EA/43-4/2019) and met the guidelines of the Animal Hygiene and Food Control Department, Ministry of Agriculture, Hungary and the European Communities Council Directive recommendations for the care and use of laboratory animals (2010/63/EU).

For brain surgeries and transcardial perfusions, anesthesia was given to rats by intramuscular injection of a ketamine (50 mg/kg)/xylazine (4 mg/kg) (Richter Gedeon) cocktail. Animals were decapitated or were perfusion-fixed with 4% paraformaldehyde in 0.1 m PBS, pH 7.4. Brains were removed quickly, frozen and stored at −80°C. Perfusion-fixed brains were cryoprotected in 20% sucrose solution before freezing.

### Immunohistochemistry (IHC)

For IHC, standard chromogenic or fluorescent immunohistochemical methods were applied (Table 1) using serial coronal 50-µm-thick free-floating sections. When ISH and IHC were combined, 20-µm-thick slide*-*mounted sections were used. Primary antibodies were applied after serum (1% BSA or 3% NDS, supplemented with 0.1% Triton X-100) and endogenous peroxidase (3% H_2_O_2_, all from Merck Life Science) activity blocking steps. Secondary antibody-conjugated peroxidase activity was eliminated by using 3% H_2_O_2_ solution supplemented with 0.01% Na-azide. Microwave treatment (5 min) was performed in 0.1 m citric acid (pH 6.0, Merck) for antigen retrieval, prevention of nonspecific cross reactions of antibodies, and/or for blocking HRP enzyme activity (Tóth and Mezey, 2007). Multiple detections were performed sequentially. The following reagents were used:

**Table 1.** Summary of the immunohistochemical reactions*^a^*

Figure	Primary/host	Detection
1 *A*	1.*^b^* PrRP/rabbit 1:10,000 2. MCH/rabbit 1:10,000	Biotinylated goat anti-rabbit IgG 1:1000, EA-HRP 1:2000 1. DAB-Ni 2. DAB
1 *B*	1.*^b^* PrRP /rabbit 1:10,000 2. TH/mouse 1:200	1. Biotinylated goat anti-rabbit IgG 1:1000, EA-HRP 1:2000, FITC tyramide 2. AlexaFluor-594-donkey anti-mouse IgG, 1:500
1 *C*	1. MCH/goat 1:500 2.*^b^* PrRP/rabbit 1:10,000 3.*^c^* TH/mouse 1:1000	1. HRP-donkey anti-goat IgG 1:1000, Pacific Blue tyramide (from TSA KIT T20920) 2. TSA KIT T20925 with goat anti-rabbit-HRP and AlexaFluor-594 tyramide 3 TSA KIT T20912 with goat anti-mouse-HRP and AlexaFluor-488 tyramide
1 *E*	1. MCH/rabbit 1:1000, or orexin/rabbit 1:1000	Biotinylated goat anti-rabbit IgG 1:1000, EA-HRP 1:2000, DAB
4*A*,*B*	1.*^b^* PrRP/rabbit 1:10,000	Biotinylated goat anti-rabbit IgG 1:1000, EA-HRP 1:2000 1. DAB
4 *D*	1. cFos/rabbit 1:20,000 2.*^b^* MCH/goat 1:500 3. PrRP/rabbit 1:10,000	1. TSA KIT T20925 with goat anti-rabbit-HRP and AlexaFluor-594 tyramide 2. AlexaFluor-647-donkey anti-goat IgG, 1:500 3. Biotinylated donkey anti-rabbit IgG 1:1000, EA-HRP 1:2000, FITC tyramide
5 *C*	1. MCH/rabbit 1:1000 2. intracellular biocytin	1. AlexaFluor-488-donkey anti-rabbit IgG, 1:500 AlexaFluor-568-SA, 1:500
6 *F*	1.*^b^* cFos/rabbit 1:20,000 2. PrRP/rabbit 1:10,000	Biotinylated goat anti-rabbit IgG 1:1000, EA-HRP 1:2000 1. DAB-Ni 2. DAB
6 *H*	1.*^b^* PrRP/rabbit 1:10,000	TSA KIT T20925 with goat anti-rabbit-HRP and AlexaFluor-594 tyramide
6 *I*	1. MCH/goat 1:500	AlexaFluor-647-donkey anti-goat IgG, 1:500

*^a^*EA, Extravidin; SA, streptavidin.

*^b^*Microwave pretreatment in 0.1 m citric acid (pH 6.0, Merck) for 5 min.

*^c^*Preincubation in 3% H_2_O_2_ solution containing 0.01% sodium azide in PBS for 15 min.

#### Primary antibodies

Primary antibodies are as follows: rabbit anti-cFos (catalog #ABE 457, Merck); anti-pro-MCH (catalog #sc-28931, raised in rabbit or sc-14509, raised in goat, Santa Cruz Biotechnology); rabbit anti-Orexin A (aa:14-33, catalog #R-104-100, Novus Biologicals); rabbit anti-PrRP (catalog #H-008-52, Phoenix Pharmaceuticals); and mouse anti-TH (catalog #MAB318, Millipore).

#### Detection reagents

Detection reagents include the following: AlexaFluor-488-conjugated Donkey Anti-Rabbit IgG (catalog #A21206), AlexaFluor-594-conjugated Donkey Anti-Mouse IgG (catalog #A21203), AlexaFluor-647-conjugated Donkey Anti-Goat IgG (catalog #A32849), AlexaFluor-568-conjugated streptavidin (catalog #S11226), and Tyramide Signal Amplification (TSA) Kits (catalog #T20912, T20920, T20925) (Fisher Scientific; Invitrogen); biotinylated donkey anti-rabbit IgG (catalog #711-065-152) and peroxidase-conjugated Donkey Anti-Goat IgG (catalog #705-035-003) (Jackson ImmunoResearch Laboratories, Izinta Trading); biotinylated goat anti-rabbit IgG (catalog #BA-1000, Vector Laboratories); DAB (catalog #D12384), extravidin-horseradish peroxidase (catalog #E2886) (Merck); and Fluorescein Tyramide Reagent Pack (catalog #SAT701B, PerkinElmer Life and Analytical Sciences).

### Analysis of PrRP and TH coexpression in the varicosities in the rat hypothalamus

Serial coronal sections from perfusion-fixed naive rats (*n* = 2) were triple-immunolabeled for MCH, PrRP, and TH (see above). Confocal *z*-stack images were acquired bilaterally through a 63× lens objective (see Imaging) from three sections per animal containing the highest density of MCH cells. The *z* separation was adjusted to 3 µm to avoid capturing the same varicosity twice. The number of PrRP^+^ varicosities were determined in the perifornical area (PFA) (20 *z* stacks) and the lateral hypothalamus (LH) (17 *z* stacks) in the single optical sections (1 AU, ROI size: 135 × 135 µm^2^). TH positivity of the same profiles was analyzed in a separate channel, using the multipoint tool in the ImageJ 1.32j software (Wayne Rasband; National Institutes of Health). The percentage of PrRP-TH coexpression was calculated for each optical section and used for the statistical analysis.

### ISH

Radioactive ISH was used for localization of the PRLHR and Neuropeptide FF receptor 2 (NPFF2R) mRNAs (*n* = 2 rats), and for measuring PrRP mRNA levels in sleep-deprived animals. Hybridizations were performed using serial coronal 12-µm-thick fresh frozen and 20-µm-thick perfusion fixed sections, as described previously (Tóth et al., 2008) using [^35^S]UTP-labeled (Per-Form Hungaria) antisense riboprobes. The probes were generated by *in vitro* transcription (MAXIscript Kit, Fisher Scientific) according to the manufacturer's instructions using the following rat cDNA sequences: PrRP (10-240 bs, GenBank AB015418) and PRLHR (189-636 bs, GenBank NM_139193) cloned into pBC KS^+^ vector (Addgene), as well as NPFF2R (112-796 bs, GenBank AF268900.1) cloned into pGemT Easy vector (Promega). The specificity of the cDNA fragments was verified by sequencing and assessed by BLAST screening of the rat genome (https://blast.ncbi.nlm.nih.gov/Blast.cgi). For signal detection, the sections were apposed to a BAS-MS imaging plate (Fuji PhotoFilm) for 5 d. The signals were read out by a Fujifilm FLA-8000 Image Analyzer. The sections were dipped into Kodak NTB autoradiographic emulsion (Agar Scientific), exposed for 5 d (PrRP ISH) or 6 weeks (PRLHR and NPFF2R ISHs), developed using Kodak Dektol developer and Fixer (Sigma-Aldrich), and were counterstained with Giemsa (Sigma), except when processed for IHC before ISH signal detection.

### Brain slice electrophysiology

Whole-cell patch-clamp experiments were conducted using acute rat brain slices from 30-d-old rats (*n* = 20) to evaluate the effect of PrRP 12-31 (3.5 µm, #P7357, Merck) on MCH neurons and to test the effect of PrRP on the NA (10 µm, #A9512, Merck) evoked responses. Rats were anesthetized by 1% Isoflurane (Baxter) 1-2 h after lights on. After decapitation, the brain was removed and 250 µm thick coronal slices containing the DLH (bregma −1.43 mm) were generated with a Leica VT-1000S vibratome (Leica Microsystems) in ice-cold, reduced sodium aCSF equilibrated with 95% O_2_ and 5% CO_2,_ (composition in mm as follows: saccharose 205, KCl 2.5, NaHCO_3_ 26, MgCl_2_ 5, NaH_2_PO_4_ 1.25, CaCl_2_ 1, glucose 10). The upper part of the slice was dissected just above the mammillothalamic tract to avoid influence of thalamic neurons expressing PRLHR (Ibata et al., 2000). Then the slices were incubated in aCSF (composition in mm as follows: NaCl 130, KCl 3.5, NaHCO_3_ 26, MgSO_4_ 1.2, NaH_2_PO_4_ 1.25, CaCl_2_ 2.5, glucose 10) for 1 h at room temperature. Recordings were conducted in aCSF at 33°C using an Axopatch-200B patch-clamp amplifier, Digidata-1322A data acquisition system, and pCLAMP 10.4 software (also used for analyses of data, Molecular Devices) under a BX51WI IR-DIC Olympus microscope. The patch electrodes (OD = 1.5 mm, thin wall, Hilgenberg) were pulled with a Flaming-Brown P-97 puller (Sutter Instruments), the resistance was 2-3 mΩ. The intracellular pipette solution contained the following (in mm): K-gluconate 130, KCl 10, NaCl 10, HEPES 10, MgCl_2_ 0.1, EGTA 1, Mg-ATP 4, Na-GTP 0.3 and biocytin (1.5 mg/ml, all from Merck).

Cells in the PFA were injected with hyperpolarizing/depolarizing square-step-pulses (duration: 900 ms, amplitudes: −30 and 30 pA, respectively) in current-clamp mode. MCH neurons were identified by their characteristic ion current responses (van den Pol et al., 2004) and by *post hoc* IHC for MCH and detection of biocytin. Ion currents were recorded in voltage-clamp mode at −70 mV holding potential. Resting potentials (V_rest_) were recorded in current-clamp mode at 0 pA holding current in the absence/presence of TTX (660 nm, Bio-Techne R&D Systems). TTX was added 10 min before recordings started and then it was continuously present in the aCSF bath solution. Measurements started with initial control recordings. Either PrRP 12-31 or NA (3.5 µm, #P7357 and 10 µm, #A9512, respectively, Merck) was added in single boluses into the recording chamber. When added together (PrRP + NA), NA was applied immediately after PrRP. Consecutive applications of NA and PrRP + NA were performed, when V_rest_ returned to the baseline. When the α2-adrenergic receptor antagonist yohimbine (2 µm, #1127, Bio-Techne) or the GABA(A) receptor antagonist picrotoxin (100 µm, #1128, Tocris) was applied, they were continuously present in the bath solution from the 10th min before NA administration. Slices were fixed in 4% paraformaldehyde overnight and processed for fluorescence detection of MCH and biocytin.

### Brain surgeries

#### Implantation of EEG electrodes

Stainless-steel screw electrodes were implanted over the left frontal cortex (stereotaxic coordinates: 2.0 mm lateral to the midline and 2.0 mm anterior to bregma) and over the left parietal cortex (2.0 mm lateral to midline and 2.0 mm anterior to λ) for frontoparietal EEG recordings. An occipital electrode as a ground was also implanted. A pair of EMG electrodes (stainless-steel spring electrodes covered by silicon rubber) was placed into the neck muscles.

#### Intracerebroventricular (ICV) cannulation

A polyethylene guide cannula (0.58 mm inner diameter, Intramedic PE-50, Clay Adams, Thomas Scientific) was inserted into the right lateral ventricle under stereotaxic control (−0.8 mm caudal to bregma, 2.0 mm lateral to midline, 4.0 mm ventral to the surface of the skull). EEG electrodes were implanted at the same time, when needed. Rats were allowed to recover and were handled for at least 7 d.

### EEG recording and analysis

The effects of ICV-injected saline and different doses of PrRP12-31 (1.6, 4.0, and 10 nmol/5 µl, Merck) on the sleep–wake stages were investigated. Injections were given to awake rats at the beginning of the light phase (lights on at 10:00 A.M.) in a crossover design with 3 d washouts between the treatments (*n* = 8). EEG recordings were compared among the different doses of PrRP and saline.

Rats were placed and kept individually during the study in glass recording chambers (35 × 35 × 40 cm), while attached to a flexible recording cable to allow free movement. The recorded EEG/EMG signals (Vital Recorder software, Kissei Comtec) were amplified (5000 and 10,000 times for EEG and EMG, respectively), and were subjected to analog filtering (<0.5 Hz and >60 Hz). Analog/digital conversion was performed at a sampling rate of 128 Hz. During the whole period of the recordings (24 h), rats were undisturbed, and had access to food and water *ad libitum*. Sleep-EEG analysis was performed in 10 s periods (epochs) using the automatic detection function of Sleep Sign for Animal software (Kissei) based on conventional criteria (Kantor et al., 2004) followed by visual assessment by experienced EEG scorers.

The following sleep–wake stages were differentiated; wakefulness: EEG signal is characterized by low-amplitude activity at β (14-29 Hz) and α (10-13 Hz) frequencies, with high EMG and intense locomotor activity; non-REM sleep (NREMS): EEG is characterized by high-amplitude slow cortical waves (0.5-4 Hz) interrupted by spindles (10-12 Hz), with reduced EMG activity and minimal motor activity; REM sleep (REMS): EEG is characterized by low-amplitude and high-frequency activity with regular theta waves (5-9 Hz), accompanied by silent EMG and motor activity with occasional twitching (Kantor et al., 2004). Power spectral analysis was computed for consecutive 10 s epochs at the frequency range of 0.5-60 Hz (fast Fourier transformation routine, Hanning window, frequency resolution: 0.25 Hz). Adjacent 0.25 Hz bins were summed into 1 Hz bins. Bins are marked by their upper limits (2 Hz refers to 1.25-2.00 Hz). The values of consecutive epochs, separately in wakefulness, NREMS, and REMS were averaged for the first 60 min after treatment. Epochs that contained artifacts or transition between sleep–wake stages were excluded from the power spectral analysis.

### Sleep deprivation

REMS deprivation (SD) was performed using the flower pot method, as described previously (Vas et al., 2013). Rats (250-350 g) were sleep-deprived for 72 h starting at lights on (at 10:00 A.M.) on small (SP, diameter: 6.5 cm) or large platforms (LP, diameter: 13 cm) surrounded by water or kept in their home cages (HC) as controls. REMS is eliminated on SP because rats fell into the water because of muscle atony during REMS, whereas LP enables REMS. Thus, LP-kept rats serve as stress controls. Platform sizes and rat weights were chosen based on our previous work (Kitka et al., 2009) and other studies (Maloney et al., 1999; Verret et al., 2003; Machado et al., 2004) validating the method. After 72 h, subgroups of SD rats were allowed to have recovery sleep (2 h) (SPR, LPR) in their HCs.

Rats were killed and the brains were used for quantitative ISH (5 groups, *n* = 5/group) or IHC (5 groups, *n* = 3/group). Additional animals were used for EEG analysis of the recovery sleep (3 h, SPR and LPR groups, *n* = 6/group). Bodyweight change and food intake during the 72 h were measured for the cohort used for PrRP mRNA and protein analysis (*n* = 8/group). EEG recordings during the sleep recovery period were compared with the undisturbed baseline recordings of each rat (1-3 h after lights on, under the same conditions).

### Measurement of PrRP mRNA expression levels in SD animals

Fresh frozen brains were cut into 12-µm-thick serial coronal sections in a cryostat, mounted onto Superfrost UltraPlus slides (Fisher Scientific), and stored at −80°C. PrRP mRNA was localized in the sections by radioactive ISH providing a linear relationship between the signal intensity and the mRNA expression level (Chen et al., 2012). Optical densities (mean gray values) of the individual nuclei were measured from the storage phosphor recordings in the A1 and A2 cell groups and between 14.04 and 14.70 mm, as well as in the DM between 3.36 and 4.08 mm caudal to bregma (Paxinos and Watson, 2007) with Image J 1.32j software. Data were determined in three and two consecutive sections in the brainstem and the hypothalamus, respectively, selected from the centers of the investigated areas. The same nuclei were evaluated by applying the same settings both across the sections and across the animals. Background values were measured in parallel and subtracted. In the A1 and DM, where optical densities showed significant differences among the groups, we determined mRNA expression levels also within the individual cells from darkfield microscopic images of autoradiographic emulsion-coated sections as described previously (Tóth et al., 2008). Microphotographs were captured through a 10× objective lens on an Olympus BX60 microscope (Olympus Hungary) from one representative section per animal (see Imaging). The silver grains were selected using the threshold tool in the ImageJ software. The amount of the silver grains over the individual cells was evaluated within identical ROIs and expressed as the area (pixels) covered by grains (Wittmann et al., 2015). In all cases, the signal threshold was set to be >3 times the mean value of two or three background samples measured in the surrounding region. All measurements were performed bilaterally.

### Counting of PrRP-immunopositive cells in SD animals

PrRP-immunolabeled cells were visualized on serial coronal sections using DAB as chromogen. Microphotographs were captured through a 10× objective lens on an Olympus microscope (see Imaging). Cells were counted bilaterally in three sections showing the strongest signals in A1 and A2 cell groups, as well as in one or two sections in the DM using the touch count tool in the ImageJ 1.32j software.

### Counting of MCH and cFos-immunopositive cells

Cells were visualized on serial coronal sections using Ni-DAB for cFos and DAB for MCH as chromogens. cFos, MCH, and double-labeled cells were counted bilaterally in four sections within 100 × 300 µm^2^ ROIs, placed over the PFA or LH using the touch count tool in the ImageJ 1.32j software.

### LHe paradigm

The LHe was performed as described previously (Otrokocsi et al., 2017) with slight modifications. Automated soundproof shuttle-boxes interfaced with a computer running Med-PC IV software (Med Associates) were used for the experiment. The shuttle-boxes consisted of two identical compartments and were equipped with photobeam sensors, stimulus light, a tone generator, stainless-steel grid floor, and a guillotine door between the compartments. The paradigm consisted of a training and a test session. On the morning of the experimental day, rats were placed individually in the left or right compartment of the shuttle-box with the guillotine door closed and left to explore for 5 min. The training session lasted for 40 min. Rats in the shocked group (*n* = 10) received 120 trials of 5 s cue (light and tone stimuli) followed by an inescapable (closed door), uncontrollable electric foot shock at 0.4 mA with random duration ranging from 5 to 15 s and unpredictable intershock intervals. Control animals (*n* = 5) did not receive the cues and foot shocks but were otherwise treated similarly. The test session started 7 d later by returning the animals to their previously assigned compartment. All rats received 30 trials of 5 s cues during which the guillotine door was open, followed by an unexpected and escapable foot shock (0.4 mA intensity, 10 s duration with random intershock intervals). Escape during stimulus or escape during shock was scored if the animal crossed the door during cue presentation or the 10 s shock, respectively. Escape latency was measured. Escape failure was scored on the animal not crossing the door during the trial. Animals were classified as being LHe-resilient or LHe-susceptible (“helpless”) by applying the *k*-means clustering (*k* = 2) method (Marques et al., 2022). The analysis was applied on the escape failure and escape latency data pairs (*n* = 15). The results were validated by applying the mean + 2 SDs criterion for the escape failure data. Bodyweight and food intake were measured immediately before the training and the test sessions, so the bodyweight change, and food intake were calculated over the 7 d (between the two sessions). Fecal discharge during sessions was counted. Rats were decapitated 24 h after testing between 11:00 A.M. and 12:00 A.M. Trunk blood was collected, the plasma was separated by centrifugation and stored at −80°C for corticosterone RIA (Zelena et al., 2003). The brains were frozen and processed for microdissection and subsequent RT-PCR.

### Microdissection of rat brain tissue

Fresh frozen brains were cut into 300-µm-thick slices in a cryostat and mounted on untreated glass slides (Fisher Scientific). The regions containing the DLH, DM, A1, and the A2 cell groups were identified using a head magnifier, with reference to the Rat Brain Atlas (Paxinos and Watson, 2007). The areas were removed from the sections bilaterally using sterile punch needles with an inside diameter of 0.7 mm. The sections were kept frozen in the cryostat throughout the whole procedure. The samples were collected in RNase/DNase-free tubes (Eppendorf) containing NucleoZOL (Macherey-Nagel), homogenized and stored at −80°C.

### RIA

Corticosterone was measured in 10 µl unextracted plasma, as described previously (Zelena et al., 2003). Assay sensitivity was 1 pmol. The intra-assay coefficient of variation was 7.5%. All the samples were measured in one assay.

### Lipopolysaccharide (LPS) treatments

Rats were treated with intraperitoneal injections of LPS (1 mg/BW kg, O55:B5, Merck) or pyrogen-free saline. Animals in Cohort 1 were killed for IHC (Table 1) 3 or 24 h (*n* = 3/group) and for RT-PCR 3 h (*n* = 5/group) after treatments. Cohort 2 was assessed in the forced swim test (FST) 24 h after LPS (LPS: *n* = 6, control: *n* = 5).

### Quantitative evaluation of the immunofluorescence PrRP and MCH signals

Sections immunostained for PrRP and MCH from control and 24 h LPS-treated animals were selected and matched according to their rostro-caudal levels. Confocal images from the PFA and LH were acquired through a 10× lens objective (see Imaging). Measurements were performed using the ImageJ 1.32j software. The PrRP fibers were separated from the background using the threshold tool. Density of the fibers was determined as the area (pixels) covered by fibers within a 100 × 300 µm^2^ ROI, placed over the PFA or LH. The total corrected cellular fluorescence in the MCH-immunostained neurons was calculated by measuring the integrated density of the cell and subtracting the background (area of measured cell × mean fluorescence of background readings) (McCloy et al., 2014). The cell bodies were outlined, and the relevant parameters were measured along with three adjacent background readings using the same outline. Only in focus-plane cells (20 cells/image) were evaluated. Data obtained from bilateral measurements of two PrRP and one MCH-immunostained sections from each animal were used for the statistical analysis.

### FST

Rats were placed individually in a cylinder (diameter: 18 cm, height: 61 cm) filled (up to 30 cm) with water at 27°C-28°C, during the light phase between 10:00 AM and 2:00 PM (Porsolt et al., 1978; Slattery and Cryan, 2012). Tests (6 min) were performed 24 h following a training session (15 min). PrRP (4 nmol) was given ICV to rats 30 min before the test session. LPS was given to rats in a separate experiment right after the training session. Behavior of the animals (*n* = 6 and *n* = 5 for treatment and control [saline] groups, respectively) was recorded by a camcorder and analyzed later by an observer blinded to the treatment groups using Solomon Coder software (https://solomoncoder.com). During the test, the time spent floating (immobility), struggling, and swimming was determined. Floating was scored when the general activity of the animal was minimized to occasional and small movements of legs or tail necessary to keep the nose above the water. Struggling was considered when the animal was permanently breaking the water surface by intense movements of forepaws usually directed against the walls. Swimming was defined as making active swimming motions, more than necessary to merely keep the head above water (Mlynarik et al., 2007). Immobility (floating) time (%) during the test was rated as passive coping behavior. Each animal was dried and returned to its cage after sessions. Bodyweight and food intake between the sessions were measured.

### Human subjects and microdissection of human brain tissue

DLH tissue samples from postmortem control (*n* = 7) and suicidal (*n* = 8) male subjects were provided by the Human Brain Tissue Bank (Semmelweis University, Budapest, Hungary). We included only males in the study to avoid possible sex bias in the data (Tóth et al., 2008) and to be consistent with our animal studies. Clinical information and permission from the patient or the next of kin for the use of the brain tissue for research purposes had been previously received. The medical history of the subjects was obtained from medical or clinical records, and from pathologic and neuropathological reports. Demographic characteristics and available relevant clinical and neuropathological information of the cohort are provided in Table 2. The tissues were stored at −80°C until dissection and later kept frozen throughout the whole procedure. Tissue samples were removed from the brains at the rostral mammillary level from 1.0- to 1.5-mm-thick coronal sections cut by an electric slicer. The regions were identified using the human brain atlas (Mai et al., 2015) and a stereomicroscope with prechilled stage. The samples were removed by using sterile punch needles with inside diameter of 1.0 mm (Palkovits, 1973), collected in RNase/DNase-free tubes (Eppendorf) and stored immediately at −80°C and used for RT-PCR analyses.

**Table 2.** Demographic, clinical, and postmortem characteristics of the control and suicide subjects*^a^*

Case no.	Age (yr)	Sex	Postmortem interval (h)	Cause of death	Neuropathology	Sample
Control						
1	49	Male	2	Cardiac insufficiency	Negative	LHA
2	52	Male	4.5	Myocardial infarction	Negative	LHA
3	54	Male	2	Myocardial infarction	Negative	LHA
4	55	Male	1	Myocardial infarction	NA	LHA
5*^b^*	55	Male	6	Myocardial infarction	Negative	LHA
6	73	Male	6	Myocardial infarction	NA	LHA
Mean ± SEM	56.3 ± 3.5		3.6 ± 0.9			
Suicide						
1	31	Male	8	Hanging	NA	LHA
2	35	Male	6	Hanging	Negative	LHA
3*^b^*	40	Male	10	Hanging	Negative	LHA
4	41	Male	4	Hanging	Negative	LHA
5	42	Male	2	Hanging	NA	LHA
6	47	Male	6	Hanging	Negative	LHA
Mean ± SEM	39.3 ± 2.3*		6 ± 1.2			
Control						
1	49	Male	2	Cardiac insufficiency	Negative	DHA
2	54	Male	2	Myocardial infarction	Negative	DHA
3	55	Male	1	Myocardial infarction	NA	DHA
4*^b^*	55	Male	6	Myocardial infarction	Negative	DHA
5	73	Male	6	Myocardial infarction	NA	DHA
6	81	Male	5	Ischemic heart failure	NA	DHA
Mean ± SEM	61.2 ± 5.2		3.7 ± 0.9			
Suicide						
1	35	Male	6	Hanging	Negative	DHA
2*^b^*	40	Male	10	Hanging	Negative	DHA
3	41	Male	4	Hanging	Negative	DHA
4	47	Male	6	Hanging	Negative	DHA
5	62	Male	2.5	Hanging	NA	DHA
6*^b^*	65	Male	10	Hanging	Negative	DHA
Mean ± SEM	48.3 ± 5.1		6.4 ± 1.3			

*^a^*The subjects were without known treatment with psychotropic medications except Patient 7 (risperidone, donepezil-hydrochloride, paroxetine all prescribed for dementia). Tissue samples were obtained from the DHA and LHA.

*^b^*Subjects with blood alcohol test. All test results were negative.

*p < 0.05, compared with control group (Mann–Whitney *U* statistic = 10.000 T = 74.000); *n* = 6/group.

Samples were collected in accordance with the Ethical Rules for Using Human Tissues for Medical Research in Hungary (HM 34/1999) and the Code of Ethics of the World Medical Association (Declaration of Helsinki) and approved by institutional ethics committees of the Semmelweis University. Human brain microdissection procedures were approved by the Regional and Institutional Committee of Science and Research Ethics of Scientific Council of Health (34/2002/TUKEB-13 716/2013/EHR, Budapest, Hungary) and the Code of Ethics of the World Medical Association (Declaration of Helsinki). The study was performed in the course of an approved study by the Ethical Committee of the Semmelweis University.

### RT-PCR

Total RNA was isolated using a GeneJET RNA Purification Kit. RNA quality and quantity were determined with a NanoDrop 2000 Microvolume Spectrophotometer and in the human samples also by examining the ribosomal RNA bands on agarose gels. cDNAs were prepared using a High Capacity cDNA Reverse Transcription Kit. Amplifications were performed on an ABI StepOnePlus Real-Time PCR system using TaqMan Universal Master Mix II and commercially available TaqMan assays (Table 3). (All above were purchased from Fisher Scientific.) Expression levels were normalized to GAPDH in the rat samples and to acidic ribosomal protein P0 in the human samples. Samples were measured in technical parallels. Relative fold changes were calculated by the comparative CT method (2^−ΔΔCt^). Amplifications were not detected in the no-template and a reverse transcriptase minus reactions.

**Table 3.** Catalog numbers of the Fisher Scientific TaqMan assays used for RT-PCRs*^a^*

Target	Human	Rat
PRLHR	Hs00244685_s1	Rn01411250_s1
NPFF2R	Hs01003259_m1	Rn00576846_m1
PrRP	—	Rn00573653_m1
pro-MCH	—	Rn00561766_g1
TNFα	—	Rn01525859_g1
GAPDH	HS99999905_m1	Rn01775763_g1
RPLPO	HS99999902_m1	—

*^a^*RPLP0, Ribosomal protein large P0.

### Imaging

Light microscopic examinations were performed using an Olympus BX60 microscope equipped with a SPOTXplorer digital CCD camera (Diagnostic Instruments). Pictures were taken using UPlanFL 4×/0.13, 10×/0.30, or 20×/0.50 objectives. Confocal images were acquired on an LSM 780 confocal laser-scanning microscope (Zeiss). For PrRP and MCH quantitative measurements, pictures were captured using a 10×/0.45 M27 objective with pinhole size 6.5 and 1 AU, respectively. MCH coexpression with biocytin was investigated using 63×/1.4 Oil DIC M27 objective with pinhole size 1.47 AU. For the quantitative analysis of PrRP and TH coexpression and for the illustrative pictures of the MCH neurons, single optical sections were captured using a Plan-Apochromat 63×/1.4 Oil DIC M27 objective with *z* separation of 3 and 1 µm, respectively. In case of the MCH staining, the stacks were *z*-projected in the ImageJ 1.32j software. Contrast and sharpness of the illustrative images were adjusted in Adobe Photoshop CS 8.0. Multipanel figures were assembled in Adobe Illustrator CS3.

### Experimental design and statistical analysis

The statistical evaluations were performed using Sigmastat 3.5 application (Systat Software). The size of the experimental groups was determined based on the literature of the field and our previous experience. Each experiment presented in the paper was repeated in multiple animals (*n* = 3-8/groups, see relevant sections). In all experiments, relevant animals were allocated to experimental groups randomly. Data analysis was performed blinded to experimental groups in all cases where the results would have been biased otherwise (in case of more than one treatment groups and manual data analysis). Statistical significance was accepted when *p* < 0.05. Data are expressed as mean ± SEM, except ΔCt data that are shown using box-and-whiskers plots. Paired or unpaired Student's *t* tests (two-sided) were used for the comparison of two groups with normal distribution of data; otherwise, we applied Mann–Whitney *U* statistics. When more than two groups were compared, one- or two-way ANOVA or repeated-measures ANOVA were conducted, depending on the experimental setup. Pearson's correlation was used to examine correlations with normal distribution of the data.

## Results

### Both medullary and hypothalamic PrRP neurons project to the DLH

**Figure 1.**
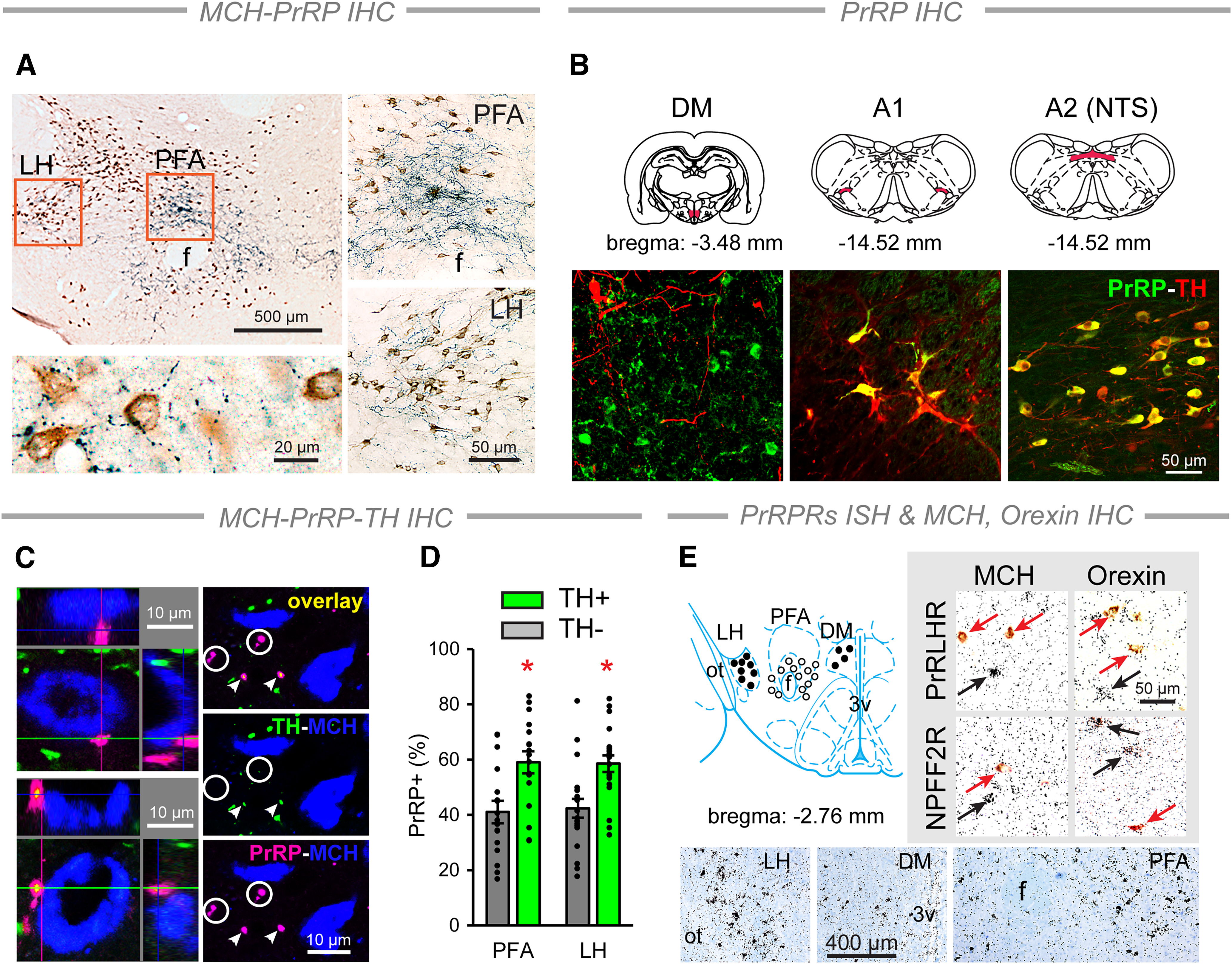
Anatomical relationship between PrRP and MCH neurons in the DLH. ***A***, Double IHC. Top left, Dense PrRP fiber (blue) network surrounds the MCH cells (brown) mainly in the PFA and LH. Right, The PFA and the LH (framed on ***A***) at higher magnification. Bottom left, Multiple PrRP^+^ axonal varicosities contact the perikarya and dendrites of MCH neurons. ***B***, Noradrenergic and non-noradrenergic PrRP cell populations. Top, Location of the PrRP cells (red) in the hypothalamic DM, and in the A1 and A2 (within the NTS) NA cell groups. Double immunolabeling of TH (red) and PrRP (green) producing cells confirms the catecholamine content of medullary PrRP cells (yellow) (Chen et al., 1999; Tóth et al., 2008). PrRP cells in the DM lack TH. ***C***, Confocal images of triple-labeled sections. Left, Both TH^–^ (top) and TH^+^ (green, bottom) PrRP^+^ terminals (magenta) form close contacts with MCH (blue) neurons. Right, TH^–^ (circles) and TH^+^ (arrowheads) axonal varicosities of PrRP^+^ fibers were analyzed in separate channels. ***D***, Quantitative analysis of the different types of PrRP^+^ profiles in the DLH. Error bars indicate mean ± SEM. Two-way ANOVA, TH: *F*_(1,70)_ = 22.46, *p* < 0.001. Student-Newman-Keuls multiple comparison test, TH^+^ versus TH^–^: *p = 0.001, *n* = 20 (PFA) and *p = 0.002, *n* = 17 (LH). ***E***, Top left, Location of cells expressing PrRP receptors (PrRPRs): NPFF2R (closed circles) and PRLHR (open circles). Bottom, NPFF2R-expressing cells in the LH and DM as well as PRLHR-expressing cells in the PFA detected by radioactive ISH. The autoradiography shows black silver grain conglomerations over the positive cells. The sections were counterstained by Giemsa (blue). Top right, MCH- and orexin-immunopositive neurons (red arrows) do not express PRLHR and NPFF2R (black arrows). f, Fornix; ot, optic tract; 3v, third ventricle. The schematic drawings of the coronal sections are from the Paxinos atlas (Paxinos and Watson, 2007).

The interaction between PrRP and MCH neurons requires an appropriate anatomic basis; therefore, we first performed morphologic studies. Our IHC findings revealed that PrRP^+^ axonal terminals distribute within the DLH. Numerous PrRP^+^ axonal terminals established close appositions with MCH^+^ neurons. This was seen mostly within two areas of the DLH, namely, the PFA and the LH (Fig. 1*A*). Confocal microscopy analysis confirmed the catecholamine content (TH positivity) of the medullary PrRP cells, which were located in the A1 and A2 cell groups. In A2, they were distributed in the nucleus of the solitary tract (NTS) (Chen et al., 1999; Tóth et al., 2008). PrRP neurons in the DM were, however, negative for TH (Fig. 1*B*). MCH-, PrRP-, and TH-triple-labeled sections showed the existence of intimate contacts between the A1/A2- and DM-originated PrRP^+^ varicosities and MCH^+^ neurons (Fig. 1*C*, left). When counting 711 and 579 PrRP^+^ varicosities in the PFA and LH, respectively, ∼60% of the varicosities contained TH in both investigated areas (Fig. 1*C*,*D*).

To see whether MCH neurons express PrRP receptors, we performed ISH combined with IHC. PRLHR mRNA-expressing cells were restricted to the PFA. NPFF2R, which mediates some effects of PrRP (Ma et al., 2009), was detected in the dorsal periventricular region in the DM and in a narrow field of the rostral LH adjacent to the optic tract (Fig. 1*E*, left and bottom). Importantly, the receptor-expressing cells were negative for MCH, which excludes the possibility that the intimate contacts between PrRP^+^ and MCH^+^ neuronal elements are direct synaptic contacts. Orexin cells, which are also a major cell population of the DLH (Kitka et al., 2011; Vas et al., 2013), did not express PrRP receptors either (Fig. 1*E*, top, right).

### ICV PrRP injection promotes sleep

**Figure 2.**
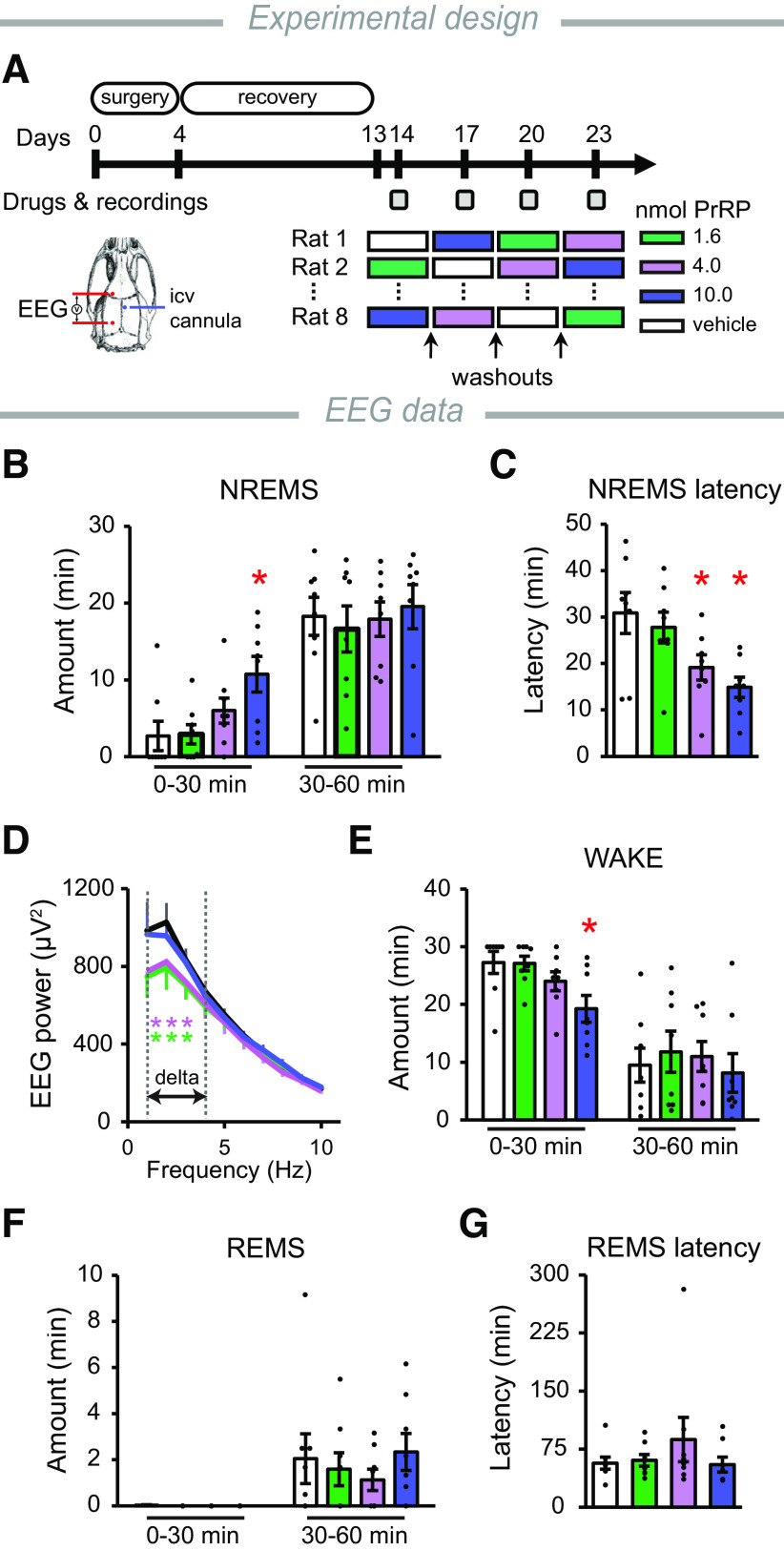
Effects of PrRP on EEG parameters. ***A***, Schematic and timeline of the experiment. Frontoparietal EEG was recorded on the left (red). Different doses of PrRP, or vehicle (saline), were injected intracerebroventricularly (ICV) through a chronically implanted cannula (blue) into the right ventricle, at the beginning of passive (light) phase using a crossover design. Gray squares represent the days when treatments and recordings were performed. ***B***, The amount of NREMS. Zero is the time point of the drug administration. One-way repeated-measures ANOVA 0-30 min, *F*_(3,21)_ = 5.14, *p* = 0.008. ***C***, NREMS latency. One-way repeated-measures ANOVA, *F*_(3,21)_ = 6.56, *p* = 0.003. ***D***, EEG δ power (1-4 Hz) during the first hour after injections. Two-way repeated-measures ANOVA, treatment × frequency interaction: *F*_(9,63)_ = 2.86, *p* = 0.007. ***E***, Time spent awake. One-way repeated-measures ANOVA, *F*_(3,21)_ = 5.09, *p* = 0.008. ***F***, The amount and (***G***) the latency of REMS. For *post hoc* analysis, Bonferroni multiple comparison tests were performed. *p < 0.05 versus vehicle; *n* = 8/group. Error bars indicate mean ± SEM.

Since there is a strong relationship between stress, sleep, and mood (van Dalfsen and Markus, 2018; Steiger and Pawlowski, 2019), and the DLH is a critical site of sleep–wakefulness regulation (Bonnavion et al., 2016), next we examined the effect of ICV PrRP administration on the sleep–wake cycle. PrRP (0, 1.6, 4, and 10 nmol) injection at the beginning of the passive phase (Fig. 2*A*) elicited an acute sleep-promoting effect by increasing the amount of NREMS and decreasing NREMS latency. Both effects ceased within 30 min and were significant with the higher doses of PrRP only (Fig. 2*B*,*C*). Lower doses of PrRP (1.6 and 4.0 nmol) also affected the quantitative EEG spectra during NREMS by reducing the power of the δ (1-4 Hz) frequency range (Fig. 2*D*). The amount of wakefulness diminished gradually along with the increasing doses of PrRP (Fig. 2*E*). The amount of REMS was nearly zero in the short period of PrRP's effects; therefore, the effects of PrRP on REMS and REMS latency could not be investigated using this experimental setup (Fig. 2*F*,*G*).

### PrRP expression is associated with sleep–wake stages

**Figure 3.**
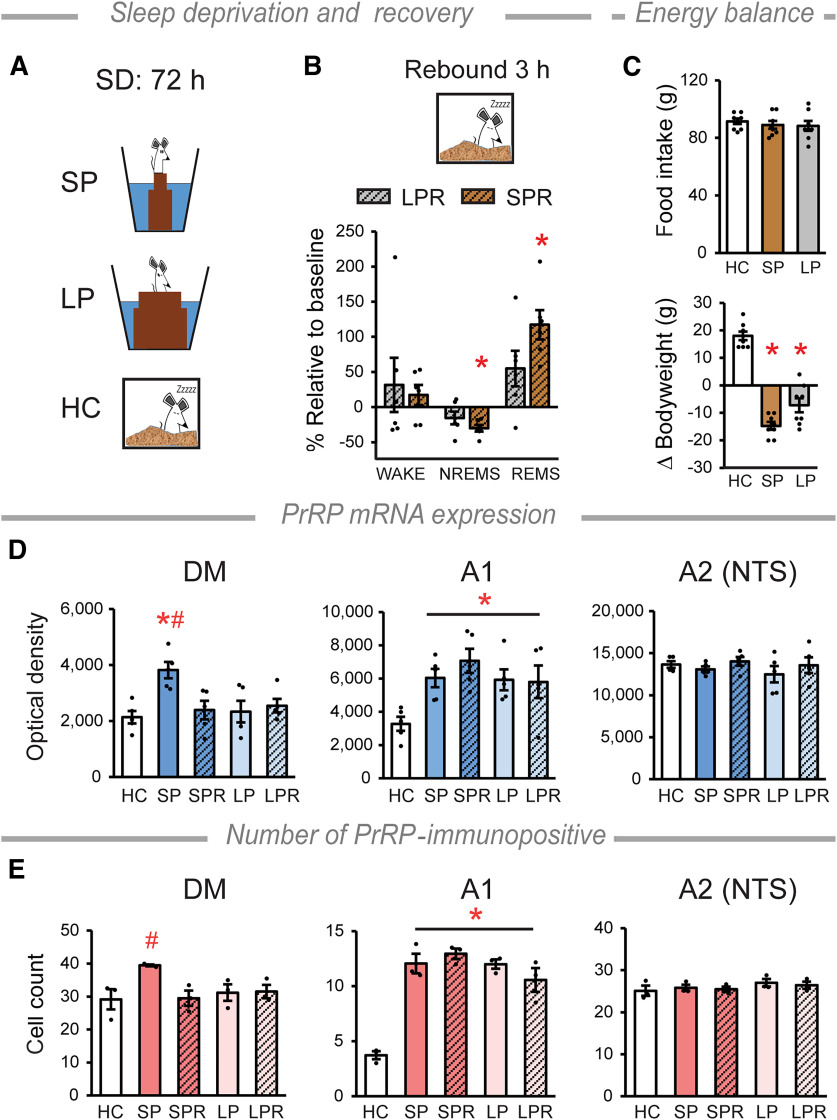
PrRP expression varies with the amount of sleep. ***A***, Schematic of SD method. Rats were placed individually on round platforms surrounded by water. REMS is eliminated on SP because rats fell into the water because of muscle atony during REMS, whereas LP enables REMS. Thus, LP-kept rats serve as stress controls (Maloney et al., 1999; Machado et al., 2004; Kitka et al., 2009). ***B***, Sleep parameters during the recovery sleep. The amount of NREMS decreases, while the amount of REMS increases significantly in the SP rebound (SPR), but not in the LP rebound (LPR) group, compared with baseline recorded for each rat. Two-way repeated-measures ANOVA, vigilance state × pot interaction: *F*_(2,15)_ = 31.6, *p* < 0.0001, NREMS *p < 0.0001 and REMS *p = 0.0002; *n* = 6/group. ***C***, Food intake (top) and bodyweight change (bottom) during SD. Kruskal–Wallis test, H_2_ = 17.19, *p* < 0.001, *p < 0.05 versus HC; *n* = 8/group. ***D***, PrRP mRNA expression levels in the DM, the A1 and A2 (NTS) cell groups. Graphs represent the quantitative results measured as optical densities of the ISH signals. DM: one-way ANOVA, *F*_(4,20)_ = 4.96, *p* = 0.006, SP versus HC *p < 0.01, SP versus all the other groups ^#^*p* ≤ 0.01. A1 cell group: one-way ANOVA, *F*_(4,20)_ = 4.16, *p* = 0.013, all groups versus HC *p < 0.05. NTS: no alterations were detected, *n* = 5/group. ***E***, Number of PrRP-immunopositive cells. DM: one-way ANOVA, *F*_(4,10)_ = 3.54, *p* = 0.048, SP versus SPR and LPR ^#^*p* < 0.05, SP versus HC and LP *p* = 0.053 and *p* = 0.061, respectively. A1 cell group: one-way ANOVA, *F*_(4,10)_ = 28.11, *p* < 0.001, all groups versus HC *p < 0.001. There was no change in the NTS. *n* = 3/group. Error bars indicate mean ± SEM. For multiple comparisons, the Holm–Sidak method (***B***) and the Student-Newman-Keuls (***C–E***) test were used.

To challenge MCH neurons that are known to be involved in promoting REMS (Bandaru et al., 2020), we used the flower pot SD paradigm, which eliminates REMS completely, and enhances the REMS pressure (i.e., the need for REMS, Fig. 3*A*) (Maloney et al., 1999; Verret et al., 2003; Machado et al., 2004). During the recovery sleep following 72 h SD, REMS was recovered in the SPR rats, by producing twice as much REMS compared with the baseline, whereas the amount of NREMS decreased. The LPR group revealed no change in the sleep parameters versus the baseline (Fig. 3*B*).

Despite consuming a similar amount of food to HC rats (Fig. 3*C*, top), both SP and LP rats lost weight compared with HC animals (Fig. 3*C*, bottom). This effect was independent of the size of the platforms.

**Figure 4.**
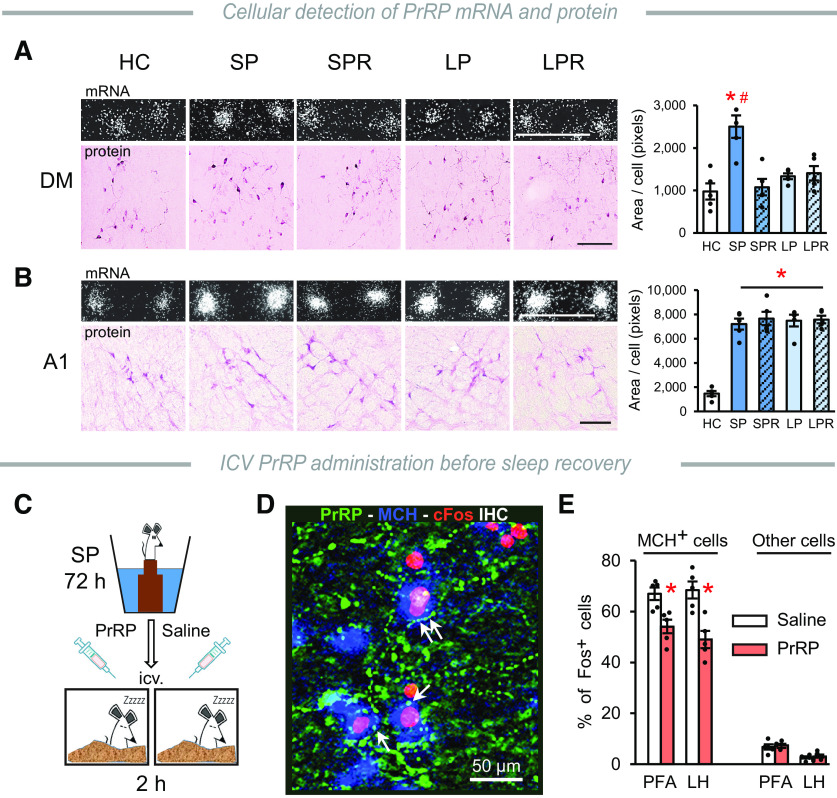
Cellular changes after SD. ***A***, ***B***, Detection of PrRP mRNA and protein in the cells in the DM and the A1 cell group, respectively. Top, left, Darkfield pictures: autoradiography of PrRP mRNA-labeled cells. The density of the silver grains (white spots) over the positive cells changes in proportion to the amount of mRNA. Bottom, left, Immunohistochemical detection of PrRP^+^ neurons. Scale bars, 100 µm. Right, Graphs represent the results of the mRNA level measurements at cellular level. Data are expressed as the area covered by silver grains over the individual cells. One-way ANOVA, DM: *F*_(4,24)_ = 10.53, *p* < 0.001, A1: *F*_(4,24)_ = 40.15, *p* < 0.001, Student-Newman-Keuls multiple comparison test, *p < 0.001 versus HC, ^#^*p* < 0.001 versus all other groups; *n* = 5/group. ***C***, PrRP (4 nmol/5 µl) or saline (5 µl) was administered ICV into SP-deprived rats prior of recovery sleep. ***D***, MCH (blue), cFos (red), and PrRP (green) triple fluorescence IHC showed close contacts between PrRP^+^ fibers and MCH^+^ neurons, which were activated by REMS rebound. ***E***, Percentages of MCH^+^ and non-MCH^+^ neurons exhibiting cFos signal in the PFA and LH in animals treated with PrRP, or saline. Two-way ANOVA, effect of treatment: *F*_(1,16)_ = 28.73, *p* < 0.001; *n* = 5/group. Student-Newman-Keuls multiple comparison test, *p < 0.01 versus saline. Error bars indicate mean ± SEM.

The SP- but not the LP-induced SD increased the amount of PrRP mRNA in the DM, and this was normalized during recovery (Fig. 3*D*). Additionally, both SP and LP conditions elevated PrRP mRNA levels remarkably in the A1 cell group, which remained high during the recovery sleep. Neither the SP- nor the LP-induced SD altered the expression of PrRP mRNA in the NTS (Fig. 3*D*). PrRP protein levels showed parallel alterations in the relevant experimental groups to the PrRP mRNA levels (Figs. 3*E*, 4*A*). Measurements at the cellular level showed that changes in the expression of PrRP mRNA in both the DM (Fig. 4*A*) and the A1 cell group (Fig. 4*B*) were because of the elevated expression of the PrRP mRNA within the individual cells.

### PrRP inhibits REMS-active MCH neurons *in vivo*

Our flower pot experimental data provided evidence for the active participation of the endogenous PrRP in the sleep–wake regulation. Additionally, it provided a useful experimental setup for the functional investigation of the morphologically established circuits between PrRP and MCH neurons. Evoking rebound sleep after SD by the flower pot method is a powerful way to activate the majority of MCH neurons in the DLH, which can be detected by cFos IHC (Verret et al., 2003; Kitka et al., 2011). To see whether PrRP affects the function of REMS-active MCH neurons, SP rats were injected with PrRP (4 nmol, ICV), or vehicle just before recovery sleep (Fig. 4*C*). We found that PrRP fibers frequently formed close contacts with cFos^+^/MCH^+^ neurons (Fig. 4*D*). In the PrRP-treated rats, the ratio of cFos^+^/MCH^+^ neurons was significantly lower compared with controls in both the PFA and LH (Fig. 4*E*, left). The percentage of other types of cFos^+^ cells was unaffected by the PrRP treatment (Fig. 4*E*, right).

### PrRP hyperpolarizes MCH neurons and greatly increases the effect of NA *ex vivo*

**Figure 5.**
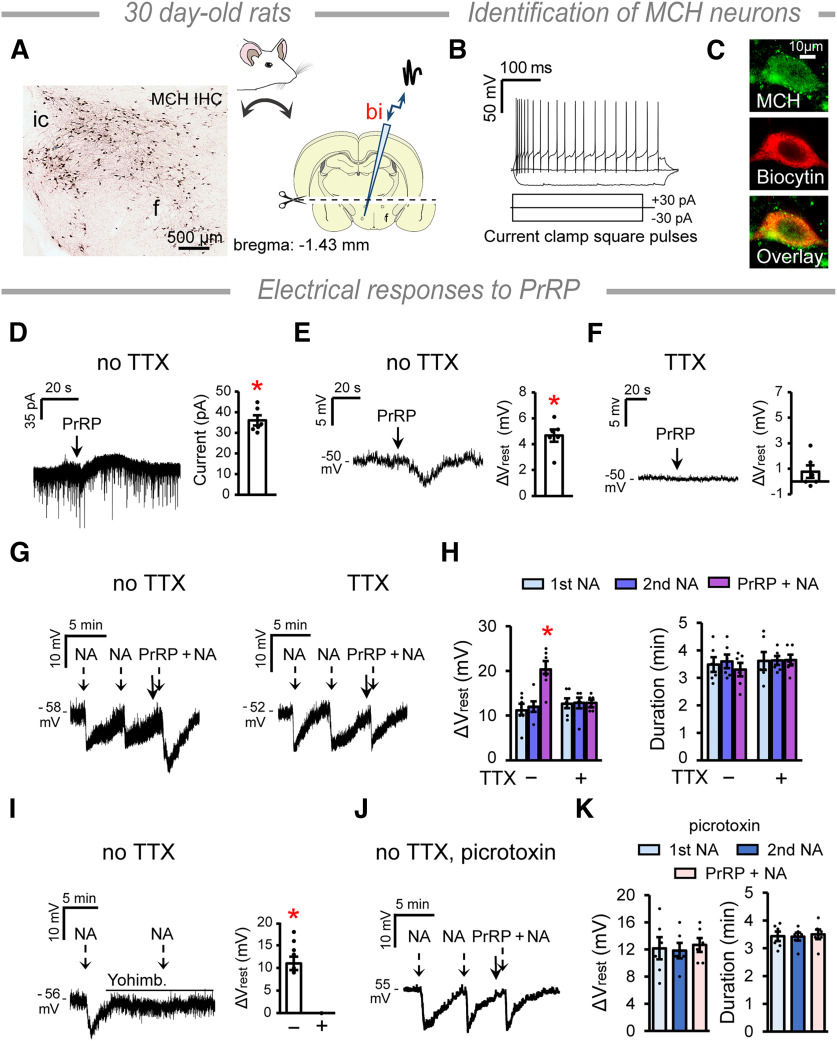
PrRP inhibits the MCH neurons. ***A***, Left, Immunohistochemical detection of MCH neurons in 30-d-old rats. Right, Schematics (Paxinos and Watson, 2007) of the brain slice preparation for patch-clamp recording. Acute coronal slices containing the hypothalamus were used with the thalamus, and the cortex removed. Neurons in the PFA were patched using a biocytin (bi) filled electrode. ***B***, Electrophysiological identification of the MCH neurons was performed by their response to current steps (van den Pol et al., 2004). ***C***, Identity of the measured cells was confirmed by *post hoc* IHC. ***D***, PrRP (3.5 µm) elicits a transient outward current in MCH neurons in the absence of TTX. Student's *t* test (paired), *t* = 14.745, df = 5, *p = 0.000. ***E***, Membrane potential changes (ΔV_rest_) of MCH neurons in response to PrRP in the absence of TTX. Student's *t* test (paired), *t* = 9.785, df = 5, *p = 0.000. ***F***, There is no response to PrRP in the presence of TTX. ***G***, ΔV_rest_ in response to repeated administration of NA (10 µm, dashed arrows), and after pretreatment with PrRP (solid arrow) in the absence (left) and presence of TTX (right). ***H***, Graphs summarize the effects of PrRP on the NA-induced responses. One-way repeated-measures ANOVA. ΔV_rest_ no TTX: *F*_(2,10)_ = 32.6, *p* < 0.001, Holm–Sidak multiple comparison tests, PrRP+NA versus first and second NA, *p < 0.0001. ***I***, ΔV_rest_ in response to NA in the absence (–) and presence (+) of the α2-adrenergic receptor antagonist yohimbine (2 µm, horizontal line above the recording). Student's *t* test, *t* = 8.900, df = 5, *p = 0.000. ***J***, ΔV_rest_ in response to repeated administration of NA (dashed arrows), and after pretreatment with PrRP (solid arrow) in the presence of the GABA(A) receptor antagonist picrotoxin (100 µm) and absence of TTX. ***K***, Graphs summarize the effects shown in ***J***. Error bars indicate mean ± SEM. *n*_cells_ = 6 for all except *n*_cells_ = 4 in ***F***. One or two cells/animal were measured in independent experiments.

To characterize the observed effect of PrRP on MCH neurons, we performed whole-cell patch-clamp experiments using brain slices from 30-d-old rats. At this age, the MCH neuronal system is fully developed (Risold et al., 2009), and the MCH cell population appears similar to those in adults (Fig. 5*A*, left). MCH neurons in the PFA (Fig. 5*A*, right) were identified by their characteristic electrophysiological responses to current steps (Fig. 5*B*) as follows: (1) adaptation of firing during the depolarizing step; (2) absence of spontaneous spikes at resting state; (3) absence of rebound depolarization at the end of the hyperpolarizing step; and (4) absence of sag at the beginning of the hyperpolarizing step (van den Pol et al., 2004). Identity of the measured MCH neurons was also confirmed by *post hoc* IHC (Fig. 5*C*).

PrRP (3.5 µm) evoked an outward current in the MCH cells, starting within 15 s after its application (I_max_: 36.06 ± 2.45 pA, duration: 28.00 ± 4.67 s) (Fig. 5*D*). PrRP hyperpolarized MCH neurons in the absence of the voltage-gated Na-channel blocker TTX with a 4.66 ± 0.48 mV change in the resting potential (ΔV_rest_) and with a duration of 27.42 ± 3.71 s (Fig. 5*E*). This effect was not observed in the presence of TTX (Fig. 5*F*).

We also investigated the interaction between PrRP and NA. As expected (van den Pol et al., 2004), NA (10 µm) hyperpolarized the patched MCH neuron repeatedly (Fig. 5*G*, left, Fig. 5*H*). When PrRP (3.5 µm) was added just before the third application of NA, the response was almost doubled. Meanwhile, the mean durations of the triggered V_rest_ changes were unaltered (Fig. 5*G*, left, Fig. 5*H*). In the presence of TTX (Fig. 5*G*, right, Fig. 5*H*), the response to NA was similar to that observed in the absence of TTX. PrRP did not modify the NA-triggered response in the presence of TTX (Fig. 5*G*, right, Fig. 5*H*). The response to NA was completely blocked in the presence of the α2-adrenergic receptor antagonist yohimbine (2 µm, Fig. 5*I*) (van den Pol et al., 2004).

Next, we used the same experimental setup to identify the cell type mediating the inhibitory effect of PrRP on MCH neurons. This time, we used bath-applied picrotoxin (100 µm), a GABA(A) receptor antagonist. We assumed that PrRP acts via inhibitory interneurons in the PFA, because PRLHR is a Gq-coupled receptor (Dodd and Luckman, 2013) and is highly expressed by GABAergic cells in the reticular nucleus of the thalamus (Lin et al., 2002). Indeed, whereas NA elicited similar responses in MCH neurons than previously, PrRP failed to modify the effect of NA in the presence of picrotoxin and absence of TTX (Fig. 5*J*,*K*). Together, our findings revealed a presynaptic, GABA(A) receptor mediated, inhibitory effect of PrRP on MCH neurons, which substantially adds to the postsynaptic action of NA. The presence of close contacts between MCH neurons and PrRP varicosities may therefore suggest that PrRP receptors are located at the GABAergic terminals innervating MCH cells.

### Overload of PrRP neurons underlines increased passive coping in FST

**Figure 6.**
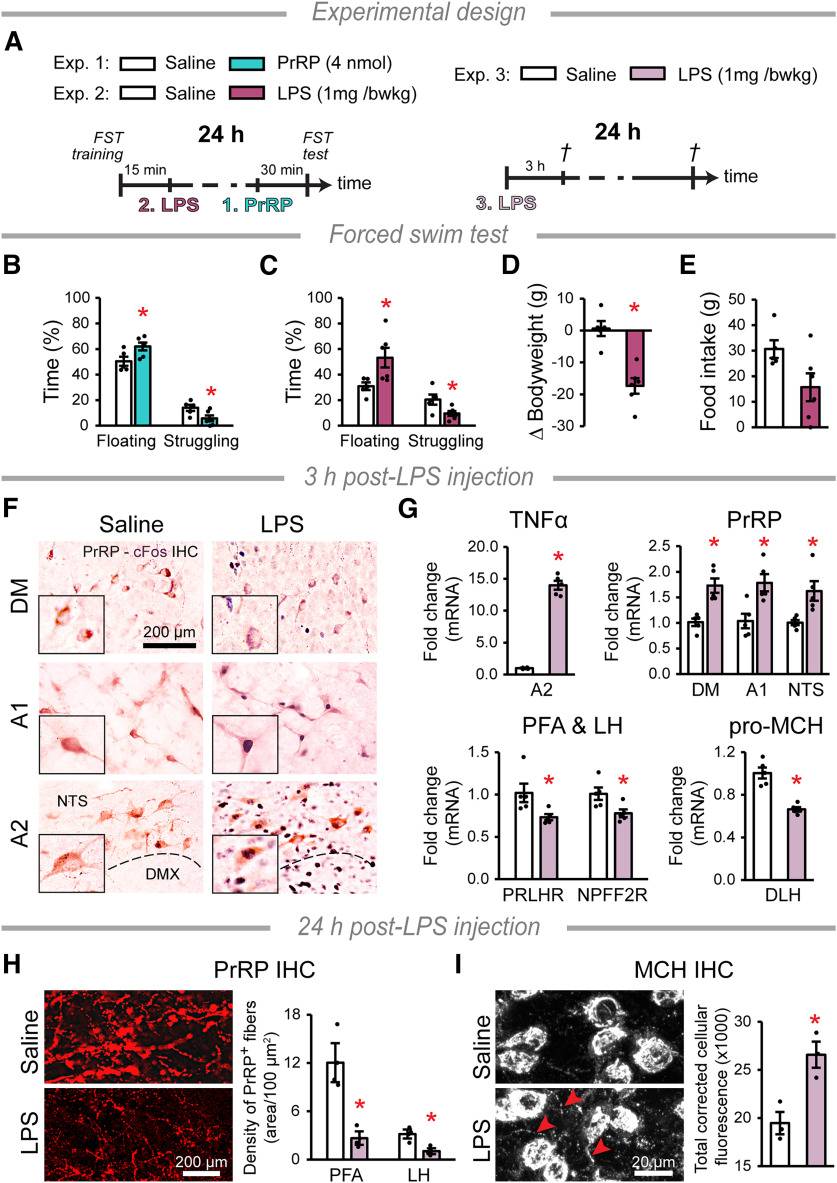
Evaluation of the inflammation-induced depression model. ***A***, Timelines of the experiments. LPS was added intraperitoneally to induce inflammation. PrRP was injected intracerebroventricularly. Control rats received saline. ***B, C***, Exp. 1. and 2, respectively: Student's *t*-tests, *df* = 9, saline: *n* = 5, treated: *n* = 6. Percentage of the time spent passively (“floating”) and actively (“struggling”) in the FST 30 min after (***B***) PrRP (floating: *p = 0.036, struggling: *p = 0.035, *t* = −2.459 and *t* = 2.484, respectively) or 24 h after (***C***) LPS treatments (floating: *p = 0.034, struggling: *p = 0.030, *t* = −2.499 and *t* = 2.576, respectively). ***D***, Bodyweight change (*t* = 5.213, *p = 0.001) and (***E***) food intake (*p* = 0.056) during the 24 h after the LPS injections. ***F***, PrRP cell (brown) activation 3 h after LPS treatment. The cFos nuclear signal (black) appears in the activated neurons. The inserts show PrRP neurons at higher magnification. DMX: dorsal motor nucleus of the vagus nerve. ***G***, Exp. 3: Student's *t*-tests, *df* = 8, *n* = 5/group. Relative mRNA levels of TNFα (*p = 0.001, *t* = 37.153, measured in the A2 cell group), PrRP (DM: *p = 0.001, *t* = 4.759, A1: *p = 0.008, *t* = 3.501, NTS: *p = 0.005, *t* = 3.814), PrRP receptors (PRLHR and NPFF2R: *p = 0.02, *t* = −2.898 and *t* = −2.890, respectively) and pro-MCH (*p = 0.001, *t* = −6.854) 3 h post-LPS treatment. ***H***, Exp. 3: Student's *t*-tests, *df* = 4, *n* = 3/group. PrRP immunopositivity in DLH axons 24 h after treatments. The image illustrates the PFA. The bar graph shows the density of PrRP^+^ fibers in the area (PFA: *p = 0.021, LH: *p = 0.034, *t* = 3.675 and *t* = 3.168). ***I***, MCH immunopositive cells in the PFA 24 h after treatments. The arrowheads point to axonal processes. The bar graph shows the total cellular fluorescence measured in the MCH neurons, Student's *t*-test, *t* = –3.989 *df* = 4, *p = 0.016, *n* = 3/group, error bars indicate mean ± SEM.

To determine whether previous exposure to PrRP (“artificial stress”) affects subsequent coping with stress, we administered ICV PrRP to rats 30 min before subjecting them to FST (Fig. 6*A*, Experiment 1). The effect of ICV PrRP on ACTH release ceases after 10 min (Seal et al., 2002), in line with the effects of PrRP on the EEG, which worn off within 30 min. Post-PrRP animals showed increased immobility (“floating”) and decreased active coping (“struggling”) behavior compared with the control group (Fig. 6*B*). The percentage of time spent swimming was unaltered (saline: 20.2 ± 4.5%; PrRP: 18.0 ± 4.5%).

Next, we used LPS treatment to induce inflammatory stress in rats (Fig. 6*A*, Experiment 2). LPS has long-term effects, first triggering a surge of pro-inflammatory cytokines and sickness behavior, leading to the development of “depression-like” behavior in the late phase (24 h) (Frenois et al., 2007; Dantzer, 2009). Compared with controls, LPS-treated rats spent more time “floating” and less time “struggling” in the FST 24 h after the treatment (Fig. 6*C*), with no alteration in the percentage of time spent swimming (saline: 30.4 ± 4.6%, LPS: 22.9 ± 7.0%). The LPS-treated rats lost weight (Fig. 6*D*), although their 24 h food intake (Fig. 6*E*) was not significantly different from controls.

We then investigated the LPS-induced effects on the brain (Fig. 6*A*, Experiment 3). A high number of PrRP cells in the A1 and A2 cell groups were cFos^+^ in the LPS-treated animals 3 h after injection (Fig. 6*F*). At the same time, the mRNA expression of the pro-inflammatory cytokine, TNFα, was strongly upregulated by LPS in the A2 cell group (Fig. 6*G*). The expression of PrRP mRNA was also increased in all PrRP-producing cell groups, while the expression of PrRP receptors and pro-MCH was decreased in DLH (Fig. 6*G*). One day following LPS treatment, PrRP immunopositivity was severely depleted in axons in the PFA and LH (Fig. 6*H*). Meanwhile, MCH protein levels were elevated in the neuronal perikarya (Fig. 6*I*).

### Normal PrRP signaling in the DLH may be protective against the development of LHe

**Figure 7.**
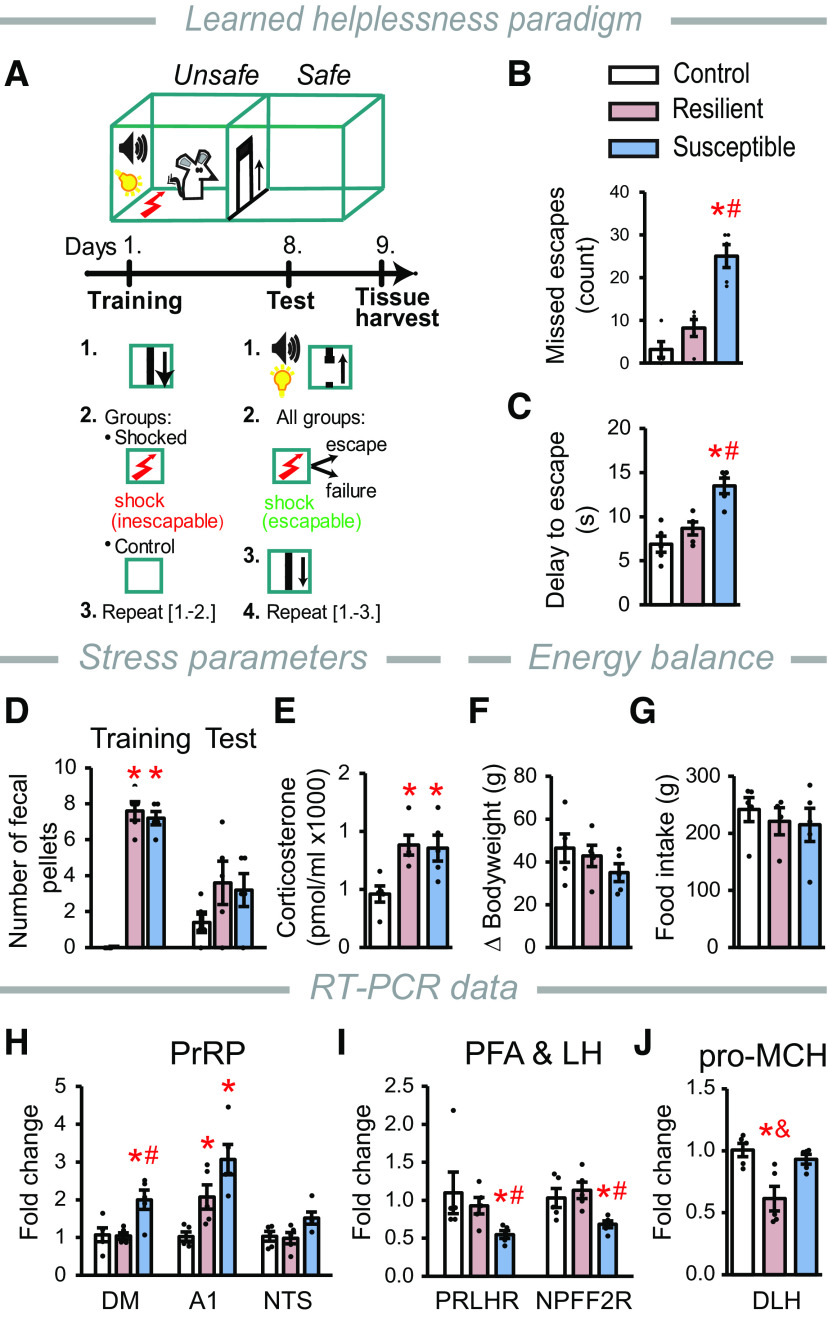
Rats with helpless behavior show altered PrRP and MCH signaling. ***A***, Schematic of the LHe paradigm. During the training, one group of animals is exposed to a series of uncontrollable and inescapable foot shock (two-chamber cage, door closed). During the test, all animals receive a series of foot shock, with prior light and sound cues provided while the door opens. ***B***, Number of missed escapes, *F*_(2,12)_ = 27.02, *p* < 0.001. *p < 0.001 versus control. ^#^*p* < 0.001 versus LHe-resilient. ***C***, Delay to escape, *F*_(2,12)_ = 16.06, *p* < 0.001. *p < 0.001 versus control. ^#^*p* = 0.002 versus LHe-resilient. ***D***, Number of fecal pellets, *F*_(2,12)_ = 137.2, *p* < 0.001. *p < 0.001 versus control. ***E***, Corticosterone levels 24 h after the test session, *F*_(2,12)_ = 6.82, *p* = 0.011. *p = 0.016, *p = 0.01 versus control, for LHe-resilient and LHe-susceptible, respectively. ***F***, Bodyweight change and (***G***) food intake between the training and the test. ***H–J***, Relative mRNA levels 1 d after the test. ***H***, PrRP: DM: *F*_(2,12)_ = 6.04, *p* = 0.015. *p = 0.025 versus control. ^#^*p* = 0.012 versus LHe-resilient. A1 cell group: *F*_(2,12)_ = 16.82, *p* < 0.001. *p < 0.004, *p = 0.001 versus control for LHe-resilient and LHe-susceptible rats, respectively. *p* = 0.053 LHe-resilient versus LHe-susceptible. NTS: *F*_(2,12)_ = 3.29, *p* = 0.073. ***I***, PrRP receptors in the DLH: PRLHR: *F*_(2,12)_ = 5.14, *p* = 0.024. *p = 0.028 versus control. ^#^*p* = 0.029 versus LHe-resilient. NPFF2R: *F*_(2,12)_ = 7.24, *p* = 0.009. *p = 0.015 versus control. ^#^*p* = 0.009 versus LHe-resilient. ***J***, pro-MCH: *F*_(2,12)_ = 8.63, *p* = 0.005. *p = 0.006 versus control. ^&^*p* = 0.006 versus LHe-susceptible. Statistics for RT-PCR were calculated from the ΔCt values. Error bars indicate mean ± SEM; *n* = 5/group. One-way ANOVA and Student-Newman-Keuls multiple comparison test were used for all statistics.

To further examine the role of PrRP in regulation of mood, we applied the LHe paradigm (Fig. 7*A*). Helpless behavior developed in 50% of the stressed rats (LHe-susceptible rats). This was clearly indicated by the number of missed escapes (Fig. 7*B*) and the escape latency (Fig. 7*C*). The escape behavior of LHe-resilient animals was normal.

Fecal discharge is a reliable marker of acute stress in rats (Bindra and Thompson, 1953). LHe-susceptible and LHe-resilient animals produced an equally high number of fecal pellets because of the inescapable shock received during the “training.” Nonshocked controls showed no signs of acute stress (Fig. 7*D*). The escapable shock received 1 week later (“test”) was a moderate level of acute stress for all animals equally (Fig. 7*D*).

Blood corticosterone concentrations were elevated 24 h after the test session in both the LHe-susceptible and LHe-resilient rats, indicating that the training induced chronic activation of the HPA axis (Fig. 7*E*). The level of chronic stress was mild, reflected by the comparable bodyweight gain (Fig. 7*F*) and food intake (Fig. 7*G*) among the groups. Importantly, although HPA activity between the LHe-susceptible and LHe-resilient rats was similar, helpless behavior developed only in the LHe-susceptible rats.

The relative expression of PrRP mRNA in the DM doubled specifically in LHe-susceptible rats (Fig. 7*H*). In the A1 cell group, PrRP mRNA level was remarkably elevated in both LHe-susceptible and LHe-resilient rats, with a near-significant difference between the two groups. The relative expression of PrRP mRNA was statistically unchanged in the NTS (Fig. 7*H*). Meanwhile, PRLHR and NPFF2R expressions in the LH and PFA were considerably downregulated exclusively in the LHe-susceptible group (Fig. 7*I*), suggesting that the development of helpless behavior could definitely be linked to the malfunction of the PrRP system in the DLH. Along with this, the expression level of pro-MCH mRNA was markedly downregulated in the LHe-resilient group, but not in the LHe-susceptible group compared with nonshocked controls (Fig. 7*J*), pointing to a possible link between PrRP signaling and MCH function.

### PrRP receptor expression is downregulated in suicidal individuals

**Figure 8.**
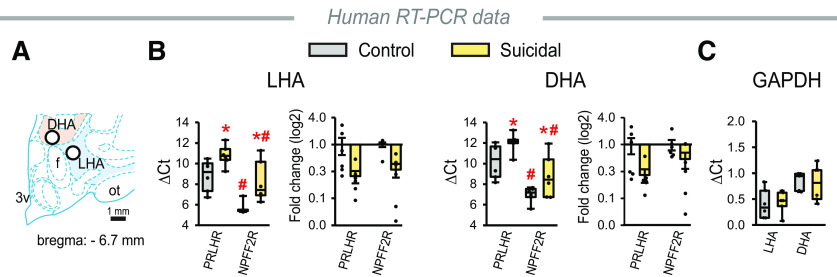
Relative mRNA expression of PrRP receptors in the LHA and DHA in control and suicidal human subjects. ***A***, Schematic of the right human hypothalamus (Mai et al., 2015). White circles represent the size and location of the areas within the LHA (blue) and DHA (beige), from which tissue samples were taken for RT-PCR. f, Fornix; ot, optic tract; 3v, third ventricle. ***B***, RT-PCR data (ΔCt and fold change) of PrRP receptors. LHA_receptors_: *F*_(1,10)_ = 27.71, *p* < 0.001. ^#^*p* < 0.001 versus PRLHR within both groups and LHA_groups_: *F*_(1,10)_ = 14.70, *p* = 0.003. *p = 0.048 versus controls within both receptors. DHA_receptors_: *F*_(1,10)_ = 23.05, *p* < 0.001. ^#^*p* < 0.001 versus PRLHR within both groups and DHA_groups_: *F*_(1,10)_ = 12.51, *p* = 0.05. *p = 0.023 and *p = 0.005 versus controls within PRLHR and NPFF2R, respectively. ***C***, RT-PCR data of GAPDH. There is no significant difference between the control and suicidal samples. Two-way repeated-measures ANOVA: ***B***, *n* = 6/group; ***C***, *n* = 5 or 6/group. One control DHA sample was excluded for technical reasons. Statistics for all RT-PCR data were calculated using the ΔCt values. Symbols label the results of the Student-Newman-Keuls multiple comparison tests. Bar graphs represent mean ± SEM. Box (indicating the first and third quartiles) and whisker (extending to the minimum and maximum data points) plots are used for showing ΔCt data.

To support the potential translational relevance of our data obtained in animal models, we measured the relative mRNA expressions of PRLHR and NPFF2R in postmortem tissue samples from suicidal and control males. Samples were obtained from the lateral hypothalamic area (LHA) and dorsal hypothalamic area (DHA) (Fig. 8*A*), which correspond to the LH and PFA in rats, respectively (Krolewski et al., 2010). Both PrRP receptors were expressed in the investigated areas with a predominance of NPFF2R mRNA. We detected a remarkable downregulation of both receptors in the suicidal group (Fig. 8*B*). We note that suicidal individuals were younger than controls in case of LHA samples (Table 2); however, there was no correlation between the ages and ΔCt values of the subjects (Table 4). We also measured the expression of another housekeeping gene, GAPDH, in the samples to verify the selectivity of our results. The expression levels of GAPDH did not differ between the control and suicidal groups in any of the investigated areas (Fig. 8*C*).

**Table 4.** Correlation analysis data between mRNA levels of the PrRP receptors (ΔCt values) and the age of human subjects*^a^*

	LHA				DHA			
	PRLHR		NPFF2R		PRLHR		NPFF2R	
	Control	Suicidal	Control	Suicidal	Control	Suicidal	Control	Suicidal
Pearson's *r*	−0.704	0.580	−0.366	0.083	−0.529	0.004	0.717	0.652
*p*	0.119	0.220	0.476	0.875	0.280	0.994	0.108	0.160

*^a^*Significance was calculated with *p* < 0.05; *n* = 6.

## Discussion

Our study points to the potential involvement of the PrRP system in the pathophysiology of stress-related mental disorders. We also show a link between failures of the PrRP signaling and dysregulation of the MCH system, known to be involved in the pathomechanism of depression, anxiety, and PTSD (Chaki et al., 2005; Lagos et al., 2011; Torterolo et al., 2015; Concetti et al., 2020). We find downregulation of PrRP receptors in DLH of suicidal individuals, highlighting the possible clinical relevance of our findings.

Emotional brain function is causally and bidirectionally related to sleep (Goldstein and Walker, 2014). Most antidepressants impact sleep, and SD may elicit antidepressant effects (Steiger and Pawlowski, 2019). Thus, getting a better understanding of common mechanisms that regulate both sleep and mood has a fundamental importance. PrRP applied at the beginning of the passive phase produced a brief NREMS-promoting effect against the high sleep pressure in awake rats, in concordance with Zhang et al. (2001). One limitation of our study is that we might have detected a longer-lasting effect of PrRP if it had been administered during the active phase, when sleep propensity is low. Interestingly, the increase in NREMS was accompanied by a decrease in δ power. Similar effects were observed after treatment with diazepam, a classic benzodiazepine drug that acts as a nonselective agonist on different GABA(A) receptor subtypes and has a subtype-specific spatial distribution (Rudolph et al., 2001; Kopp et al., 2004). Our data thus suggest that the effects of PrRP are mediated, at least in part, by GABA. Consistent with this, PrRP inhibited MCH neurons through a GABA(A) receptor-mediated mechanism in our experiments. In addition, PrRP also acts on GABAergic cells in the reticular nucleus of the thalamus (Lin et al., 2002).

Our *ex* and *in vivo* results suggest that PrRP inhibits MCH neurons and substantially adds to the inhibitory effect of NA on MCH neurons. This is in line with earlier data reporting the cooperation of PrRP and NA to elicit ACTH release (Maruyama et al., 2001). The release of neuropeptides during neuronal firing varies with time and depends also on the firing pattern of the neuron (Cropper et al., 2018). Therefore, it is possible that the PrRP to NA ratio changes in the proximity of the MCH neurons depending on the actual activity of PrRP and PrRP-NA neurons. We hypothesize that PrRP may be an ideal molecule to fine-tune the activity of MCH neurons, by enabling and maintaining it within appropriate limits.

The expression of neuropeptides is dynamic, and their release is followed by *de novo* synthesis in the cell body (Hokfelt et al., 2000). We identified a PrRP cell population in the DM, in which the expression of PrRP was upregulated (suggesting release) by SD when most of the MCH neurons are usually inhibited (van den Pol et al., 2004; Blanco-Centurion et al., 2016). However, MCH neurons are critical to promote REMS, and the number of actively firing MCH cells increases over time during NREMS and peaks during REMS (Blanco-Centurion et al., 2019). The active state of MCH neurons during REMS recovery (Verret et al., 2003; Hassani et al., 2009) was associated with the downregulation of PrRP in the DM in our study. Together with the fact that ICV PrRP injection reduced the number of cFos^+^ MCH neurons during REMS recovery, and the number of cFos^+^ MCH neurons correlates with the number of REMS episodes (Kitka et al., 2011), it is likely that PrRP is involved in the inhibition of REMS under certain challenged conditions. Since REMS-active MCH neurons regulate hippocampus-dependent forgetting of contextual fear-related memories (Izawa et al., 2019), PrRP may influence the development of stress-related mental disorders associated with hippocampal dysfunction by modulating MCH activity (Cominski et al., 2014; Wingenfeld and Wolf, 2014). In addition, the majority of REMS-active MCH neurons are also active during exploratory behavior (Blanco-Centurion et al., 2019), which changes with anxiety (Heinz et al., 2021). Further, PrRP expression was elevated in the A1 cell group in all treatment groups in the SD experiment, suggesting a stress-related reaction on the platforms. This is in harmony with the key role of the A1 cell group in the activation of the HPA axis (Buller et al., 2001; Tóth et al., 2008) that essentially modulates sleep as well (Buckley and Schatzberg, 2005).

We postulated the involvement of PrRP in the development of repeated/chronic stress-evoked mental disorders based on different types of animal models. In the FST experiment, we activated the PrRP neurons (Dodd and Luckman, 2013) 30 min after the ICV administration of PrRP. The PrRP receptors were presumably inactivated by the time of the test, since GPCRs, like receptors of PrRP, undergo a ligand-stimulated desensitization/internalization (Luttrell and Lefkowitz, 2002; Madsen et al., 2012). This happens within several minutes *in vivo*, rendering the cell to be insensitive for hours (Scherrer et al., 2006). Indeed, the acute effects of ICV PrRP were no longer detectable than 30 min (Seal et al., 2002), and previous exposure to PrRP led to impaired running stress-stimulated ACTH release (Ohiwa et al., 2007). This experiment, therefore, directly linked previous exposure to PrRP to increased passive coping, suggesting that PrRP dysfunction may underlie the development of stress disorders.

We observed a depletion of PrRP immunoreactivity in axons innervating the DLH 24 h after LPS injection in the LPS-induced depression model (Frenois et al., 2007; Dantzer, 2009). There had been a prolonged PrRP release in the area: we detected downregulation of PrRP receptors in the DLH, and upregulation of PrRP expression (Hokfelt et al., 2000; Luttrell and Lefkowitz, 2002), while most of the medullary PrRP cells were still cFos-positive 3 h after LPS injection, indicating active firing of neurons (Barth et al., 2004). Others reported that cFos positivity of neurons in the medulla was detected up to 6 h following LPS treatment (Frenois et al., 2007). After an initial decrease, MCH immunoreactivity increased 24 h after treatment. This experiment also confirmed that impaired PrRP signaling may be associated with behavioral despair.

The role of neuroinflammation in the pathophysiology stress disorders has been highlighted recently (Echeverria et al., 2016). PrRP-NA neurons in the NTS represent the first interface between the vagus-mediated peripheral signals in the brain, as vagal afferents innervate most noradrenergic cells directly (Appleyard et al., 2007). The importance of the vagus-mediated peripheral signals is further supported by the finding that stimulation of the vagus nerve has anti-inflammatory and anti-depressant effects (Liu et al., 2020).

Using the LHe model, we designated the stress-induced PrRP overload in the DLH as one of the mechanisms potentially responsible for the susceptibility to LHe. PrRP overload could also affect the function of MCH cells. Foot shock activates the medullary PrRP neurons (Morales and Sawchenko, 2003). MCH neurons are also activated by foot shock, and inhibition of this activity causes a relapse into excessive cued fear response (freezing) for weeks (Concetti et al., 2020). Therefore, if the training-induced PrRP overactivation inhibited the optimal function of MCH cells in LHe-sensitive animals, they may have exhibited an enhanced fear response during the test.

On the other hand, acute, pretest MCH treatment increased the helpless behavior of previously shocked rats compared with nonshocked controls in the LHe paradigm (Urbanavicius et al., 2019). Chronic downregulation of MCH expression in LHe-resilient animals, therefore, may have been protective against the enhanced sensitivity to MCH in the test eliciting MCH cell activation. Importantly, PrRP receptors were not downregulated in the DLH in the LHe-resilient animals, in contrast to LHe-susceptible rats. Given the role of PrRP as a potential negative regulator of MCH cell activity, it is possible that the chronic/repeated stress-induced impairment of PrRP signaling contributes to failure of downregulation of MCH in LHe-sensitive rats. Together with MCH hypersensitivity, this may lead to a reduced ability to cope with stressful situations. Since the development of helpless behavior and symptoms in the LHe model are reminiscent of those of human depression (Wang et al., 2017), PrRP receptor downregulation found in the corresponding hypothalamic areas in suicidal individuals may have a fundamental significance. Although the etiology of suicide is complex, stress-related mental disorders underlie almost all cases, with depression being at the forefront (van Heeringen and Mann, 2014; Bryan, 2016; Bachmann, 2018).

Based on our multiple correlative data, we suggest that repeated/chronic stress leads to PrRP overload, and dysfunction of the PrRP system, consequently increasing the risk of developing stress-induced mental disorders. Dysregulation of MCH activity may also contribute to the development of these mental disorders and is likely to be associated with PrRP dysfunction in the DLH. Further work is needed to verify this hypothesis.

SNRI antidepressants are widely used to treat stress-related mental disorders, although the side effects and frequent inefficacy limit their benefits (Voineskos et al., 2020). Development of antidepressant drugs with a combined effect on monoaminergic and peptidergic systems may open new directions. Compounds targeting the GPCRs, such as PrRP receptors, are optimal for this purpose and may have a great therapeutic potential in the future (Mantas et al., 2022).
